# Optimizing emergency response systems in urban health crises: A project management approach to public health preparedness and response

**DOI:** 10.1097/MD.0000000000041279

**Published:** 2025-01-17

**Authors:** Tom Nyamboga Ongesa, Okechukwu Paul-Chima Ugwu, Chinyere N. Ugwu, Esther Ugo Alum, Val Hyginus Udoka Eze, Mariam Basajja, Jovita Nnenna Ugwu, Fabian C. Ogenyi, Michael Ben Okon, Regina Idu Ejemot-Nwadiaro

**Affiliations:** a Department of Public Administration and Management, Kampala International University, Kampala, Uganda; b Department of Publication and Extension, Kampala International University, Kampala, Uganda; c Health Care and Data Management Leiden University; d Department of Public Health, School of Allied Health Sciences, Kampala International University, Kampala, Uganda; e Directorate of Research, Innovation, Consultancy and Extension (RICE), Kampala International University, Kampala, Uganda.

**Keywords:** community engagement, emergency response, ethical considerations, project management, public health preparedness

## Abstract

Effective management of health crises requires public health preparedness and response, especially in urban settings where the complexity and scope of catastrophes provide considerable challenges. The integration of project management frameworks with public health policies is highlighted in this review, which investigates the optimization of emergency response systems using a project management methodology. The adoption of cutting-edge technologies that improve real-time monitoring, predictive analytics, and resource allocation such as artificial intelligence (AI), big data, and the Internet of Things (Io-T) is one of the main topics covered. The assessment also discusses how crucial it is to take ethics into account when making decisions, how to distribute resources fairly, and how to actively engage communities to build resilience. Technological and tool innovations in project management are emphasized as critical to enhancing response times and accommodating changing circumstances. The review also emphasizes the necessity of ongoing learning and development based on prior experiences to improve preparedness tactics and overall efficacy. Public health systems can respond to urban health emergencies in a more coordinated, equitable, and efficient manner by combining these components, which will eventually improve outcomes and resilience in impacted populations.

## 1. Introduction

Community health preparedness and response are central to the safety of the populace against numerous health threats for instance, communicable diseases, disasters, bioterrorism, and chemical and nuclear attacks.^[1,2]^ Optimized readiness and response require coordinated efforts among government agencies, healthcare organizations, and Intergovernmental Organizations to ensure effective actions.^[3]^ Briton’s experiences from other nations can help understand how to set up and implement strong Public Health Response Plan.^[4]^

Consequently, the threat of bioterrorism has influenced the aspect of preparedness for bioterrorism in United States health facilities.^[5,6]^ After the anthrax attacks in 2001 that September, efforts were deployed by the U.S. government to increase its level of defense against biological weapons.^[6]^ One of them has been the establishment of the Public Health Emergency Preparedness program within the Centers for Disease Control and Prevention.^[7]^ Public Health Emergency Preparedness assists state and local health departments with funding and guidance for the development and maintenance of sound preparedness and response plans.^[8]^ These plans englobe a wide range of measures such as illness monitoring, laboratory investigations, emergency medical services, and public information and warnings.^[8]^ The case of the United States on Bioterrorism preparedness draws attention to the need for a multifactored approach that involves both health and security.^[9]^ Because of its geographical location, the country is prone to natural disasters, mainly, earthquakes.^[10]^ The lesson that the 2011 Great East Japan Earthquake brought home was the need for an effective public health response strategy.^[11]^ In response to the calamity, the central government together with local governments worked out an effective response program that included health services, counseling, and disease control measures immediately after the tragedy occurred.^[12]^ It also led to the creation of better early warning systems, and more focused public awareness of disasters amongst other things.^[13]^ The study of Japan shows the importance of continuous improvement of the readiness policies and the strength of the community for addressing natural disasters.^[14]^ The recent epidemic of the Ebola virus which happened in the Western Africa region between 2014 to 2016 from a global perspective also shows the effects of the spread of infectious diseases and the importance of global health intervention systems.^[15]^ Liberia, Sierra Leone, and Guinea faced arguably the greatest challenges in containing the virus due to the weak healthcare systems and access to healthcare.^[15]^ The people of the affected countries were not alone; the international community including the World Health Organisation (WHO), the United Nations, and many non-governmental organizations helped out handsomely.^[16]^ Those included sending out medical teams, setting up treatment facilities, and small-to-mass communitive social campaigns meant to popularize the virus and how the population could protect itself against the virus.^[17]^ The Ebola breakout planted the importance of international cooperation and the need for robust health systems to rapidly identify, isolate, and treat all contagious diseases.^[18]^

Singapore has received appreciation from the international fraternity for its early actions and measures for Coronavirus Disease 2019 (COVID-19) reactions.^[19,20]^ The nation was implementing a very effective measure that included testing, contact tracing, and strict measures on quarantine centers.^[21]^ Thus, the employment of innovations is proceeding, for instance, the use of the Trace Together application in contact tracing enabled the government to quickly identify and quarantine potential cases.^[22]^ Moreover, reliable and transparent communication with the public was another significant factor in sustaining trust and following all the necessary precautions.^[23]^ Dealing with pandemics reveals a high level of preparedness in the Singaporean healthcare system, as well as the effectiveness of the administrative approach in planning for new potential risks of ill health.^[24]^

The challenges are unique because it is easier to spread a virus, difficult to evacuate people, many types of structures present there, and many people from diverse cultural backgrounds live in the urban areas.^[25]^ Preparedness and response in public health must draw from global lessons to improve urban emergency response systems.^[26]^ The strategies and realities of different countries in containing communicable diseases are very resourceful in the enhancement of the speed of urban emergency operational systems.^[26]^ Several countries’ experiences demonstrated the relevance of the constant improvement and adaptations of the emergency preparedness of public health systems.^[27]^ Urban communities should also ensure that they periodically review their preparation mechanisms based on emerging risks and lessons learned on acts that occurred in the past.^[28,29]^ This includes funding the acquisition of the new technologies that would be employed in emergencies, training the human resources in various aspects of emergencies, and staging practice and exercises to test and improve on various responses.^[30]^ The urban emergency response systems can be made to retain the indicated capacity and efficiency by ensuring the development culture is embraced throughout the shift in handling health emergencies.^[31]^ Therefore, public health preparedness and response consists of complex and multisegmented functions that require an elaborate strategic approach as well as collaborative and effective execution. Adopting a project management model enhances health projects by providing structures and enhanced approaches in today’s societies.^[32]^

## 2. Methods

### 2.1. Literature search

A search was conducted in the various academic databases such as PubMed, Scopus, Web of Science, and Google Scholar. The following keywords were used: “project management in public health,” “urban health crisis management,” “artificial intelligence (AI) in emergency response,” “Internet of Things (IoT) for health monitoring,” “ethics in public health emergencies” and “community participation in health emergencies.” The search was restricted to articles published within the last 10 years.

### 2.2. Source identification

Sources were identified according to their titles and abstracts. The inclusion and exclusion criteria were applied as follows:

### 2.3. Inclusion and exclusion criteria

A detailed explanation of the criteria used to include or exclude studies is vital for transparency and to minimize biases:

#### 2.3.1. Inclusion criteria

- **Relevance**: The studies were required to directly examine the intersection of project management principles and public health, specifically within the context of urban health crises. This ensured alignment with the paper’s scope.
- **Quality**: Only peer-reviewed articles, high-impact case studies, and reports from accredited journals were considered. This measure was adopted to prioritize reliable and well-researched sources.
- **Focus Areas**: Studies must address at least one of the key themes: project management frameworks, technological innovations (e.g., AI, IoT), ethical considerations, resource allocation, community participation, or learning from past experiences.
- **Recency**: Priority was given to articles published within the last 10 years to reflect the latest advancements in technologies and methodologies.
- **Language**: Articles published in English or those with comprehensive English abstracts were included to ensure accessibility and consistent interpretation.

#### 2.3.2. Exclusion criteria

- **Irrelevance**: Studies not addressing the integration of project management within public health emergencies or unrelated to urban settings were excluded.
- **Low Quality**: Non-peer-reviewed sources, opinion pieces, and articles from unrecognized journals were excluded to maintain a high standard of evidence.
- **Outdated Information**: Articles older than 10 years were omitted unless they provided foundational knowledge essential to the discussion.
- **Language Barriers**: Articles in languages other than English and lacking sufficient English summaries were excluded due to interpretation limitations.

#### 2.3.3. Potential biases

- **Search Bias**: The reliance on specific databases (e.g., PubMed, Scopus, Web of Science, and Google Scholar) might exclude relevant studies published in niche journals or emerging databases.
- **Language Bias**: Restricting to English-language publications may overlook valuable insights from non-English literature.
- **Time Frame Limitation**: The ten-year restriction may exclude studies with enduring relevance but published before this period.

### 2.4. Analyzing existing public health emergency protocols

The standard measures and guidelines designed for public health emergencies are a set of concrete and coordinated steps to counter health threats, such as outbreaks of contagious diseases, natural catastrophes, and acts of terrorism (Table 1). These protocols often PRE include plans for disease reporting, resource distribution, medical crisis management, and communication with the public.^[33,34]^ However, the addition of project management frameworks can significantly enhance the quality and productivity of most of the current guidelines.^[35]^ Management frameworks like the Project Management Institute’s Project Management Body of Knowledge (PMBOK) give a systematic method of the planning and execution of projects and their control, monitoring, and appraisal.^[42]^ The application of these frameworks to the points of response in public health ensures improved efficiency in handling resources as well as dynamism in responses to the occurrence of the event.^[36]^ The incorporation of stakeholder management in the PMBOK may also help to facilitate inter-agency and inter-organization cooperation during disaster response efforts. Furthermore, due to risk management methodologies, the prospective challenges can be defined and mitigated as it offers a good reaction mechanism to organization’s project risks.^[38]^ Analyzing current practices, the deficiencies and possibilities, where the application of project management principles can provide benefit, can be identified by public health authorities.^[39–41]^ Procedures could lack adequate ways of planning and allocating resources, but the latter problem can be addressed with regard to the resource management processes described in the PMBOK. Crisis communication preparatory methods in project management communication techniques can improve communication preparations to ensure accurate and coherent messages in crisis events.^[37]^

**Table 1:** Integration of project management frameworks, specifically PMBOK, can enhance various aspects of public health emergency protocols

Aspect	Current protocols	Enhancements through project management (PM)	References
Disease reporting	Existing protocols include disease reporting mechanisms.	PMBOK can standardize reporting and ensure timely updates and monitoring.	[33–35]
Resource distribution	Protocols include resource distribution plans but may lack efficiency.	PMBOK’s resource management processes can optimize the allocation and utilization of resources.	[34–36]
Medical crisis management	Crisis management steps are outlined but could be improved.	PM frameworks ensure systematic planning, execution, and control of crisis management efforts.	[33–35]
Public communication	Communication with the public is a standard protocol.	Crisis communication techniques from PM frameworks can improve the accuracy and coherence of messages.	[34,35,37]
Inter-agency cooperation	Cooperation among agencies is essential but may be underdeveloped.	Stakeholder management in PMBOK can enhance collaboration between different agencies.	[36,38]
Risk management	Some risk management strategies are present, but may not be comprehensive.	PMBOK’s risk management methodologies help identify, mitigate, and respond to potential risks.	[38]
Identification of deficiencies	Current practices may have gaps in planning and resource allocation.	PM principles provide a framework to identify deficiencies and improve current practices.	[39–41]
Communication preparations	Preparations for crisis communication may lack structure.	PM communication techniques ensure well-prepared and coherent messaging in crisis events.	[37]

### 2.5. Case studies of successful integration in urban settings

The issue of COVID-19 in New York City is an example of how project management ideas can be integrated into emergency response plans as shown in Table 2. During one of the first and acuteness outbreaks of the disease in America, the city had to use project management approaches when organizing its response actions.^[43,44]^ The NYC COVID-19 Response Project was initiated, planned, and implemented through careful planning, effective use of available resources, and the participation of all key stakeholders to address the crisis.^[45]^ The effort included the rollout of field hospital assessments of testing capacity, and ensuring that PPEs reached different centers on time.^[46]^ Efficiencies using the project management tools and approaches provided for a well-coordinated and structured approach, making a case for the necessity of implementing such models for public health emergencies.^[55]^ Tokyo’s approach to earthquake mitigation and response is a good example of how project management best practices can be integrated into public health.^[47,48]^ Being highly prone to earthquakes this Japanese city has laid down elaborate contingency plans some of which comprise project management. The disaster management plan at the Tokyo Metropolitan Government includes detailed project schedules, resource plans, and risk management schemes.^[49]^ These guidelines helped to contain a swift and appropriate response during the Great East Japan Earthquake in 2011. This was achieved through rapidly sending out medical teams’ responses, efficient distribution of resources as well as constant communication with the public.^[50]^ The integration of these project management frameworks allowed for a continuous process, thus reducing the overall impact on public health and safety.^[56]^ To illustrate how the notions of project management are integrated into public health management in Singapore, the COVID-19 pandemic response by the country will be described.^[51]^ When drafting the response plan to the COVID-19 outbreak in Singapore, the government incorporated aspects of project management, focusing on careful planning, resource utilization, and control.^[52]^ To coordinate its response, the Ministry of Health has formulated a mechanical approach to testing, contact tracing, and implementing quarantine measures.^[53]^ Technology utilization particularly the Trace Together app was therefore part of a well-coordinated effort to involve different stakeholders such as the government, healthcare workers, and the public in general.^[54]^ This ensured an effective and quick response and minimized the spread of the virus while maintaining the confidence and trust of the public.^[54]^

**Table 2:** Case studies of successful integration of project management in urban settings

Case study	Details	Project management integration	References
COVID-19 Response – New York City	One of the earliest and most severe outbreaks in the U.S. required coordinated emergency response measures.	Project management tools ensured efficient resource use, stakeholder participation, and timely actions.	[43–46]
Earthquake Response – Tokyo	Tokyo’s approach to mitigating earthquakes involved detailed contingency plans, including project management.	Project management frameworks ensured swift responses, resource allocation, and constant public communication.	[47–50]
COVID-19 Response – Singapore	Singapore’s government implemented a mechanical approach to handling the pandemic.	Project management ensured coordinated testing, contact tracing, and quarantine measures, backed by technology use.	[51–54]

### 2.6. Role of stakeholder engagement in urban health crisis management

**Figure 1.**
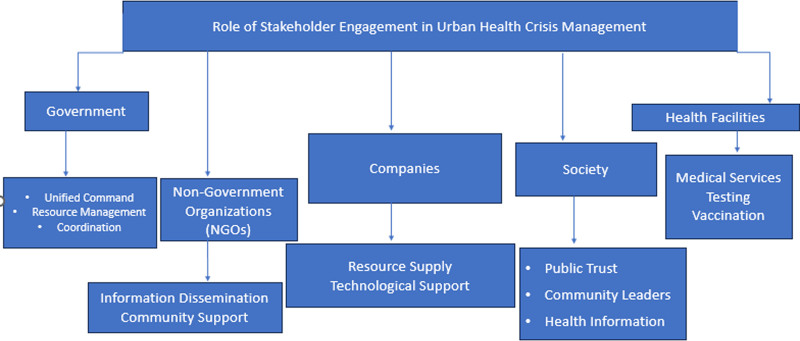
Role of stakeholders engagement in urban health crises management.

Effective coordination is important when dealing with crises that affect health in urban areas since it cements partnerships from several sectors as shown in Figure 1. Stakeholders include the government, health facilities, non-government organizations, companies, and society.^[30,57]^ Participation requires the development of clear communication structures; regular provision of correct information; and involving community leaders in disseminating health information.^[58]^ During the COVID-19 pandemic, New York City’s emergency management adopted the principle of a unified command where many activities are coordinated thus enhancing the effectiveness of the response.^[59]^ Thus, there are as many organizations involved that it is possible to manage resources, accelerate the response rate and maintain public trust in this matter.^[60]^

### 2.7. Identifying key stakeholders in urban health crises

The involvement of multi-sectoral stakeholders who have critical roles in urban health emergencies to manage health emergencies efficiently and to conduct preparedness and response and recovery operations (Table 3). Government Agencies include local, state & federal health departments, emergency management agencies as well as any other related governmental organization.^[30]^ In the context of the COVID-19 outbreak, the Centers for Disease Control and Prevention in the United States and state and local health departments had roles in managing response activities and disseminating guidance.^[61]^ Primary, secondary, and tertiary care facilities including hospitals, clinics, primary care physicians, and emergency medical services are healthcare deliverers who directly address health complications.^[62]^ Some of their responsibilities may include delivering medical services to afflicted people, managing the distribution of health-related resources, and implementing measures to improve health.^[63]^ During the epidemic, healthcare systems were involved in testing, diagnosing, treating patients with diseases, and administering vaccines.^[64]^ The Non-Governmental Organizations (NGOs), faith-based organizations and other local community-based organizations are therefore crucial in the dissemination of information as well as the provision of support services and mobilization of communities.^[65]^ These organizations often have a heightened connection with the community and may perhaps communicate pertinent messages to these marginalized groups that may be ignored by formal institutions.^[66]^ Essential service providers and pharma companies, supply chain companies, and other industries make essential contributions to ensure the availability of medicines, food, and other requirements important for sustenance.^[67]^ Such collaborations with the manufacturers of medicines proved to be helpful during the emergency in terms of quick formulation and mobilization of COVID-19 vaccines.^[68]^ The General Public is one of the key stakeholders in public health policies because the community plays a very significant role in the implementation of the said policies.^[69]^ Public participation involves informing the public with accurate information and ensuring that they are keen on following health measures.^[70]^

**Table 3:** The roles of various stakeholders in managing urban health crises and their contributions to effective emergency responses

Stakeholder	Role in urban health crises	References
Government Agencies	Involvement in managing health emergencies at local, state, and federal levels, including disseminating guidance and policies.	[30,61]
Primary, Secondary, and Tertiary Care Facilities	Directly address health complications, manage resource distribution, and implement health measures, including testing and vaccination.	[62–64]
Non-Governmental Organizations (NGOs) & Faith-Based Organizations	Disseminate information, provide support services, and mobilize communities, especially marginalized groups.	[65,66]
Essential Service Providers & Pharma Companies	Ensure availability of medicines, food, and other essentials, and collaborate on vaccine development and distribution.	[67,68]
General Public	Participate in implementing public health policies, receive and act on accurate health information, and follow health measures.	[69,70]

### 2.8. Strategies for effective stakeholder engagement and communication during crises

Effective coordination and consultation with stakeholders or interested parties on issues to do with urban health emergencies are essential.^[45]^ Measures to enhance effective reinforcement to ensure accurate, clear, and continuous communication will go a long way to enhance the impact of response efforts.^[71]^ Providing different personalities with communication channels that are tailored to the needs of different stakeholders is a good prerequisite for distributing information.^[72]^ This is accomplished by the use of technology, new media, traditional media, and word of mouth to cover the target populace as required.^[73]^ In the course of the COVID-19 epidemic, health departments used their accounts on various social media platforms and government websites to share information and recommendations.^[74]^

Therefore, this study identifies accurate and timely information to the stakeholders as one of the key components that build trust and facilitate coordination.^[75]^ This includes regular and summarized briefs, and clear reporting of the present position, risks, and management actions.^[48]^ Another aspect that was crucial to the COVID-19 was the strong practice of Singapore in terms of communication since the government delivered precise timely messages and used unambiguous language to the public.^[49]^

In this way, to reach out to the communities and ensure that a lot of the information disseminated is complied with, it is best to involve prominent figures in the community.^[76]^ Such people often have a set base of trust within the community and can also disseminate public health messages. During the Ebola epidemic, community leaders were able to spread information and encourage the use of vaccines within various areas of Africa.^[77,78]^

The general response can be enhanced by stringent structures for the human interaction of the concerned stakeholders.^[79]^ This is through the formation of task forces, working groups, and joint operation centers that foster coordination of agencies and sectors from disaster management.^[80,81]^ The establishment of a single command system by the New York City Emergency Management Department in the incident of COVID-19 is an excellent case of integration and cooperation.^[82]^ It also allows strategies to be adjusted and response measures changed in the light of feedback from the various stakeholder feedback channels.^[83,84]^ This iterative process ensures that the response is both relevant and optimized.^[85]^ The Loops have especially had their importance in the COVID-19 situation where the policies can be adjusted based on the new data and from the stakeholders.^[86]^ When crises occur, there are conflicting interests because of the different priorities, objectives and resources involved when stakeholders, e.g. governments, NGOs, private sectors, etc., do not see eye to eye.^[82]^ Because effective crisis response depends so much on being able to successfully manage these conflicts. Coordination strategies encompass such things as a common goal and transparency as well as shared resource access.^[77]^ A unified crisis framework offers the development of shared vision that all stakeholders buy in to, such as the minimum mortality rate or equitable resource distribution.^[78]^ Without role clarity, we can find ourselves with overlapping and conflicting roles.^[79]^ Collaborative forums where planning and decision making can be internally created through the multi stakeholder platform.^[82]^ Feedback from affected populations can be integrated into community engagement to make sure that strategies are equitable.^[84]^ Communication will be transparent if you want to develop a trust and reduce misinformation.^[77]^ Coordination can be done by updating regularly and have conflict resolution mechanisms among the sectors.^[83]^ Pooled resource and technology sharing between private sectors, governments and NGOs can be encouraged for resource sharing.^[84]^ Adaptive governance can also implement flexible policies and agreements by having all parties able to adjust their strategies as needed.^[85]^ Coordination is difficult due to power imbalances, different timescales, and differences in culture and politics.^[86]^ These problems must be addressed through proper mandate of equitable representation in crisis task forces, align incentives and utilize neutral mediators to lead the process and knit the gaps.^[80]^

Addressing vaccine nationalism and ensuring equitable global distribution are important and this is illustrated through a case study of coordinating during COVID-19. With COVAX, conflicts of interest were tempered as global resources were pooled and private companies were encouraged to come to the parties’ party with the promise of advanced market commitments.^[87]^

### 2.9. Resource allocation and management in public health emergencies

Effective and sustainable utilization of critical resources including medical commodities, human resources, and financial resources is very important for the achievement of the intended goals during a public health crisis.^[87]^ Various project management techniques help in managing the resources with the help of Critical Path and Resource Levelling that dictates the important tasks Crucial work sequencing and prevents over allocation of resources by leveling them.^[88]^ During the current COVID-19, the U. S Federal Disaster Management Agency (FEMA) employed these methods in supervising the distribution of PPE and vaccines; therefore, enhancing the efficiency and rapidity of the disaster response.^[89]^ Being able to distribute resources effectively contributes not only to the problems of shortage and delay but also to the improvement of the overall performance and success of emergency interventions.^[90]^

### 2.10. Project management techniques for efficient resource allocation

Optimal use of material, human, and financial resources is extremely important in managing medical supplies and staff during a public health crisis.^[91]^ Efficiency in the use of resources through project management activities can also be enhanced by the various methodologies that are available in the management of the resources.^[92]^ Techniques including Critical Path Method (CPM) and Resource Levelling are extremely helpful for the handling of complicated emergencies.^[93]^ The important Path Method enables one to identify the most critical activities, which determine the project timeline, and ensure the focus of important assets in critical areas.^[94]^ Resource Levelling is the process by which the project plan is changed to adjust the demands of resources so that they do not transform into more than they can handle. This is especially the case where the amount of available stock is rather limited, such as in emergency circumstances.^[95]^ In the course of the COVID-19 pandemic, the U. S. Federal Emergency Management Agency (FEMA) also provided leadership on the distribution of PPEs, vaccines, and other critical resources.^[96]^ After getting the distribution results to his surprise, FEMA was able to maximize its resource management and grant resources to the areas that required it most while at the same time making a point that no area was favored over the other.^[97]^ Also, the adoption of project management tools such as Microsoft Project, and Smartsheet helped in the monitoring of resource utilization, overseeing supplier chains, and the ability to make changes on the go making the process more efficient and responsive.^[98]^

### 2.11. Impact of resource management on the effectiveness of emergency response

Efficient resource management directly impacts the functionality of emergency response operations.^[99]^ Thus, by allocating resources depending on their importance and the level of their required use, they will be used optimally.^[100]^ Lack of supply management leads to delays in acquisition and shortage of resources as well as inefficient utilization of response assets, thus undermining response efforts and aggravating the crisis.^[101]^ Inadequate coordination of resources was evident in the 2014 Ebola outbreak in West Africa as inadequate medical supplies and healthcare workforce hampered the response measures and opened the virus transmission doors.^[102]^ On the other hand, during the COVID-19 response, the measures of resource intensity management, for example, a centralized approach to the delivery of vaccines and targeting the high-risk population, have contributed to an enhanced response outcome.^[101]^ For instance, the U.K.’s National Health Service (NHS) adopted a tiered system for administering the vaccine with the most vulnerable people getting the vaccines first; this played a crucial role in limiting the spread of the virus.^[102]^

### 2.12. Risk assessment and mitigation in health crisis management

Project management in health crises provides a systematic approach to assessing, analyzing, and mitigating risks.^[103]^ Tools including Risk Identification and Risk Analysis are mandatory for identifying threats like disease epidemics or supply chain disruptions and determining appropriate measures that should reduce their impact.^[104]^ In the time of COVID-19, risk management frameworks were employed in identifying some of the profound risk sources in healthcare and supply chain systems.^[105]^ This allowed for the deployment of specific actions such as the provision of additional PPE and the conceptualization of backup strategies. These strategies are complemented by specialized risk assessment procedures, including Urban Health Risk Assessment Frameworks, which consider specific urban challenges and vulnerabilities; thereby improving crisis and resilience as a whole.^[106]^

### 2.13. Applying risk management principles from project management

In the context of emergency public health response, risk management concepts originating from project management are vital for risk identification and management.^[107]^ They include a systematic approach to risk identification, assessment, and response planning. Regarding this, some approaches that may be utilized include Risk Identification, Risk Analysis, and Risk Response Planning.^[108]^ Risk assessment involves the recognition of existing risks and vulnerabilities likely to affect public health, for instance, outbreaks or natural disasters.^[11]^ Risk Analysis assesses the likelihood and impact of risks and categorizes them based on their level of priority: high, medium, or low risk.^[109]^ Risk Response Planning entails the identification of measures that can be taken to address acknowledged risks, including the development of contingency plans and resource mobilization.^[110]^ In the context of COVID-19, various risk management techniques were applied in managing various risks like supply chain disruptions and the burden exerted on the healthcare systems.^[108]^ To efficiently manage these risks, tools like the Risk Register were used to effectively assess the risks and more so the controls in place across the project.^[111]^ This strategy helped the public health organizations to come up with reaction plans that would ensure sufficient availability of medical commodities and avoid the failure of the health care system.^[112]^

### 2.14. Development of risk assessment tools tailored for urban health crises

The creation of specific procedures and frameworks for risk assessment tools for urban health crises means the establishment of a process that is going to suit the unique features of the urban area.^[113]^ Such approaches could consider parameters like population density, accessibility of infrastructure, and division into the socio-economic tiers. One of the strategies is the Urban Health Risk Assessment Framework (UHRAF) which uses GIS in mapping and analyzing risks in the urban space.^[114]^ It helps to identify such people who are at higher risk, to assess the potential of healthcare organizations in the region, and to discover which places require more attention according to the level of danger.^[115]^ Another valuable method is the Health Impact Assessment (HIA), which considers the potential impact of a health-related program on urban communities, including primary and secondary effects.^[116]^ For example, when the Zika virus was spreading across the world, cities including Rio de Janeiro already applied local approaches to risk assessment to determine which areas were most vulnerable to the virus.^[117]^ They then put measures that interfered with the reproduction of mosquitoes in those areas to curb the problem. Such techniques helped forecast threatened areas and correspondingly helped determine specific areas to focus on and minimize the outbreak’s effects. Reducing high vulnerability and improving public health outcomes therefore requires urban health authorities to develop bespoke risk assessment tools to optimize emergency response practices.^[117]^

### 2.15. Techniques for creating and managing schedules in high-pressure emergency response scenarios

Regarding time management and timetabling, these are important, especially in emergency reaction situations where time is of the essence.^[19]^ Regarding time constraints, techniques like the Critical Path Method (CPM), Gantt Charts, and Agile Project Management are works of art in handling schedules.^[20]^ Critical Chain is a way of showing how important tasks should be scheduled to avoid the overall delay of the project according to the Critical Path Method.^[21]^ It focuses on the actions that likely have a large effect on the overall delivery time of the project. Gantt Charts provide an individual with a temporal view of the project to monitor progress concerning the established time frames and proactively adjust the timelines.^[23]^ In the context of the COVID-19 epidemic, these approaches were used to manage the fast-paced adoption of testing and vaccination programs.^[37]^ Through the use of Gantt Charts, the authorities in public health were thus able to map out a good strategic plan of how to administer the vaccines to ensure that all the logistical activities matched the right schedule.^[53]^ Moreover, knowledge of Agile Project Management that focuses on flexibility and continuous improvement contributed to immediate alterations to developments and emerging circumstances and the enhancement of the effectiveness of the response.^[26]^

### 2.16. Balancing rapid response needs with long-term health outcomes

Thus, before actually planning and scheduling operations, factors such as immediate reaction required as well as long-term health impacts containment should be carefully considered.^[22]^ They include the following: Scenario Planning, Resource Optimisation, and Integrated Scheduling. Scenario planning involves the development of several strategic plans concerning the probable future occurrences in an organization to meet both the short and long-term needs.^[37]^ This enables one to avoid situations that may hinder the pursuit of organizational goals and also develop strategies that can balance short-term operations and long-term goals.^[17]^ For example, as it is with the effects of natural disasters like Hurricane Katrina, it was possible for emergency response efforts to concentrate on the effective and efficient implementation of rescue and relief operations at the same time as they sought to plan and plan for post-disaster rehabilitation and rebuilding.^[53]^ This way, through integrated scheduling, the responders were able to manage resources effectively, ensuring adequate resources for the initial response as well as for the eventual rehabilitation phase.^[33]^ Therefore, resource optimization solutions that focus on categorizing and prioritizing resources by the level of emergency needs and recovery plans will enable an organization to create a balanced balance between the immediate response and stabilizing organizational health.^[21]^

### 2.17. Quality assurance in emergency health services

However, it must be recognized that quality assurance has a vital and practical need to be installed anywhere, especially concerning emergency health services organized to develop constant quality care throughout the crises.^[7]^ The sooner the above appeals and other quality assurance factors such as standardized protocols, staff training, and performance review are embraced, the better the patient outcomes.^[8]^ For instance, when the Coronavirus outbreak started, there were some guidelines regarding the use of protective gear and the way patients should be dealt with across different medical facilities.^[12]^ Training was conducted frequently so that the healthcare workers were trained fully on the best practices to use and the performance assessments offered the quality of care to be delivered and areas of improvement as well.^[8]^ These measures were found to have significant effects on errors and patient outcomes to support the significance of comprehensive quality assurance in providing emergency health services.^[6]^

### 2.18. Implementing quality control processes in the delivery of emergency health services

Having standard measures in emergency health facilities becomes important to ensure that patients receive high-quality, safe, and consistent service in emergencies.^[29]^ Quality control measures involve compliance with company standards, practice, mastery of quality standards, and recurrent inspection of quality.^[37]^ Standardised protocols applied across a uniform system guarantee the standard application of emergency measures and, therefore, reduce deviations that could cloud medical results and care provided.^[39]^ An example is the use of standardized triage tools like ESI to prioritize patient requirements, about the level of emergency. This will allow for prioritizing the people in desperate need of medical assistance.^[49]^ Regular training ensures that the emergency professionals continually update themselves on best practices and new procedures, a factor that comes in handy, especially at a time when these disasters are constantly evolving. Performance control involves the assessment and evaluation of performance data at fixed intervals to identify what requires improvement and ensure that the standard is maintained.^[29]^ As seen in the COVID-19 crisis, many healthcare organizations employed similar quality control measures to manage a surge in patients.^[12]^ For instance, to address the reception of COVID-19 patients within the hospitals, standardized procedures had to be put in place, including the use of PPE and treatment algorithms.^[45]^ This was done to ensure that the quality and standard of care offered was the same in all the facilities.

### 2.19. Evaluating the impact of quality assurance measures on patient outcomes during crises

Evaluating the efficiency of emergency health services as well as the aspects that might require amendments can be conducted by studying the effects of quality assurance on patient outcomes.^[11]^ Care processes like adherence to clinical protocols, very frequent safety checks, and constant outcome evaluation affect the patients’ outcomes because they ensure the delivery of high-quality and risk-free care.^[53]^ Sanitary procedures and care during the Ebola outbreak were rigorous and carefully followed different measures against infections.^[44]^ This led to a drastic cut in the rate of spread of the virus among the affected patients thus increasing survival rates. Likewise, in the course of the COVID-19 epidemic, adhering to quality assurance activities, including updating treatment protocols and tracking patient outcomes, was critical in enhancing care and reducing mortality by 20%.^[15]^ The effectiveness of such measures can be evaluated by performance audits, patients’ satisfaction, and data on outcomes.^[74]^ These impact assessments are useful in assessing the utility of the interventions and guiding the enhancement process.^[13]^

### 2.20. Communication strategies in urban health crises

Project management methodologies and the use of information communication technology are applied in the handling of urban health emergencies by providing timely, accurate, and coherent information (Table 4). This can be done effectively through the application of Stakeholder Analysis and Communication Planning which help in tailoring the actual message as well as assures the adoption of identifiable procedures in disseminating information.^[13,16]^ These techniques are enhanced by technological interventions in the form of Emergency Notification Systems and Geographic Information Systems (GIS) that enable quicker notification and deployment of resources.^[9]^ Organised channels and social media were employed by cities like New York during the COVID-19 epidemic for disseminating timely information and addressing reactions. This underlines the need to incorporate project management and technological means in the communication of crises.^[12]^

**Table 4:** The key communication strategies in urban health crises

Communication strategy	Description	References
Stakeholder Analysis & Communication Planning	Tailors message to specific audiences and ensure the adoption of structured procedures for information dissemination.	[13,16]
Emergency Notification Systems	Technological systems that provide rapid notification and resource deployment during emergencies.	[9]
Geographic Information Systems (GIS)	Enables the mapping and tracking of resources and incidents, facilitating a more efficient response.	[9]
Organized Channels & Social Media	Utilized by cities like New York during the COVID-19 pandemic for timely information dissemination and public engagement.	[12]

### 2.21. Developing effective communication plans using project management principles

The importance of properly coordinated distribution of information in crises that can occur in urban areas calls for the application of project management concepts when designing communication strategies and structures.^[12]^ Some of the essential components of Project Management include Stakeholders, Communication Planning and Risk Management which are necessary for the formulation of proper communication channels.^[68]^ Stakeholder Analysis involves consideration and understanding of the needs of many organizations that are affected by the issue, the community, the health care providers, and the government among others. This tailors the flow of communication to their particular concerns and needs that are unique to each of them.^[74]^ Information management involves a strategy that outlines how and when some information is being passed to stakeholders. This encompasses identifying authorities and outlets through which the information will be communicated.^[61]^ Communication risk management comprises strategies for identifying and avoiding disinformation and ways of making sure that good information is passed on to everybody with due timeliness and precision. Some cities have developed elaborate communication plans during the COVID-19 epidemic that incorporated these guidelines.^[21]^ New York City officials use Stakeholder Analysis to engage the leadership of the community and local organizations to ensure the message is culturally appropriate and widely communicated.^[64]^ The Communication Planning strategy included regular sharing of information with the press and through other social media platforms. On the other hand, the Risk Management initiatives were about tackling disinformation about the virus and the vaccines.^[118]^ By using this orderly approach it was possible to ensure orderly flow of information and managing of the public response during the calamity.^[119]^

### 2.22. Leveraging technology for real-time information dissemination and coordination

It is critical for the quick retrieval of information and the logistics of urban medical emergencies.^[120]^ Technological systems including but not limited to Advanced Alerting Systems, GIS, and Social Networking play a significant role in enhancing communication and cooperation. Notification systems ensure the delivery of automatic messages to the general public and particularly to emergency personnel to guarantee proper and timely information relay.^[121]^ GIS offers real-time mapping of affected areas, thus aiding in the organization of resources and disease spread monitoring. Social media platforms enable the quick sharing of information and engagement of communities, which makes it possible to make frequent updates that are followed by immediate participation by various people in societies.^[76]^ During the 2020 wildfire outbreak in California, the emergency response personnel used GIS to map the spread of the fire as well as the existing evacuation corridor. Furthermore, social media was a common tool used for conveying information to the citizens and for interacting with the organs of the community.^[122]^ Similarly, social media platforms and emergency notification systems were highly useful in disseminating public health guidelines and coordinating responses across many sectors during the epidemic.^[123]^ The application of these technologies might enhance the confrontation of the municipal health authorities to manage emergencies effectively and properly to ensure that information is disseminated promptly and without mistakes.

### 2.23. Training and capacity building for emergency response teams

In this view, structuring and addressing preparedness and emergency is a significant design and implementation of effective training for the emergency response teams.^[29]^ Such programs should employ competency-based education, interdisciplinary collaboration, and role-play to provide challenging, skills-focused learning and foster a team approach.^[34]^ Here the importance of CPD is to provide the learners with up-to-date practice and technology to enhance their effectiveness in combating emerging dangers. In the times of COVID-19, constant engagement in CPD helped implement change in treatment approaches and PPE, vital for maintaining high levels of care and proper response measures.^[53]^

### 2.24. Designing and implementing training programs for emergency response teams

Ensuring there is efficient training of the emergency response teams implies that the staff is well-prepared to handle an emergency.^[9]^ To achieve a high level of experience and to enhance the components of the specific abilities, the training activities and curricula should reflect realistic scenarios, including simulations and exercises. Elements of these programs include the use of training modules covering relevant skills, the program’s interdisciplinary nature, and their scenarios.^[11]^ Skills-based training focuses on the development of some specific competencies required in emergency phases, for example, medical skills or incident management processes. Interdisciplinary training involves working with many organizations and other stakeholders to enhance cooperation in emergencies.^[21]^ Tabletop exercises are used for practicing responses of different teams inside a realistic imitation of a real-life emergency. For instance, FEMA in the United States holds training exercises like the National Level Exercise (NLE), in which several agencies’ participation is warranted in simulating crises on a large scale.^[124]^ The goal of these exercises is to evaluate and improve the capacity of a team to respond sufficiently, thus guaranteeing that the teams are prepared for a range of possible scenarios.^[17]^ Similarly, in the COVID-19 epidemic, some HC systems adopted training interventions that remain relevant in addressing the systemic and peacetime management of significant patient influxes and PPE utilization, thus emphasizing the need for practical and contemporary training.^[9]^

### 2.25. Role of continuous professional development in maintaining preparedness

However, it is important that everyone involved in the emergency response programme should be updated with the existing practice, methodological approach, tools, support model or standard and hence CPD offers an opportunity on the same.^[11]^ Moreover, Continuing Professional Development (CPD) is defined as career long learning that integrates training, education and skill enhancement activities that are required to address new challenges or enhancement of the effectiveness of the response.^[44]^ Everytime the emerging threats are revised, there are developments in the procedures and enhancements in the equipment make the responders relevant and convinced in their roles as responders.^[12]^ For instance, during the Ebola outbreak the healthcare workers had to participate in the CPD schemes more often to be aware of the latest research and trends in the treatment and prevention of the disease. In an effort to assist the healthcare professionals to become more prepared for change, WHO made it possible to update the HCAs on a continuous basis and made training material available to them.^[124]^ Likewise, in the context of the COVID-19 epidemic, it was crucial to constantly educate on the changes in the treatment guidelines, proper use of PPEs, and vaccination to adhere to high standards of care and cope with the epidemic effectively.^[11]^

### 2.26. Monitoring and evaluation of emergency response initiatives

Monitoring and evaluating emergency response initiatives are essential for assessing effectiveness and guiding improvements.^[17]^

### 2.27. Project management tools for monitoring and evaluating the effectiveness of emergency response actions

Project management tools are vital for monitoring and evaluating the effectiveness of emergency response initiatives.^[11]^ Gantt Charts, Key Performance Indicators (KPIs), and Project Dashboards are widely used tools that help track progress, measure outcomes, and identify areas for improvement.^[36]^ Gantt Charts provide a visual timeline of activities and milestones, which helps in assessing whether response actions are proceeding according to schedule.^[16]^ KPIs are specific, measurable metrics that evaluate the success of various aspects of the emergency response, such as response time, patient triage accuracy, and resource allocation efficiency.^[16]^ Project Dashboards consolidate data from various sources into a single view, enabling real-time monitoring of key metrics and facilitating quick decision-making.^[17]^ For example, during the COVID-19 pandemic, dashboards were utilized to monitor hospital capacities, track infection rates, and manage vaccine distribution, providing a comprehensive view of the response effectiveness.^[19]^

### 2.28. Developing metrics and KPIs for performance assessment

Developing metrics and KPIs is crucial for performance assessment in emergency response initiatives as they provide objective criteria for evaluating success and identifying areas needing improvement.^[50]^ Metrics should be aligned with the specific goals of the response initiative and cover various dimensions such as operational efficiency, patient outcomes, and resource utilization.^[14]^ Common KPIs include response time, which measures the speed of initial intervention, treatment success rates, which assess the effectiveness of medical interventions, and resource utilization rates, which evaluate how efficiently resources are deployed.^[18]^ For instance, during the Ebola outbreak, the World Health Organization (WHO) established KPIs to track the speed of case identification, patient treatment times, and the efficacy of containment measures.^[14]^ Similarly, in the context of the COVID-19 pandemic, metrics such as ICU bed occupancy rates and vaccination coverage were essential in assessing the effectiveness of the response and making data-driven adjustments to strategies.^[19]^ Developing and monitoring these KPIs ensures that response initiatives are effective, resources are used efficiently, and desired outcomes are achieved.^[69]^

### 2.29. Lessons learned from past urban health crises

Reviewing past urban health crises provides critical insights for improving future responses.^[5]^ For example, the SARS outbreak underscored the importance of early detection and transparent communication to curb disease spread, while the Ebola epidemic highlighted the need for stronger healthcare infrastructure and international collaboration.^[15]^ The COVID-19 pandemic further demonstrated the value of flexible, scalable response strategies and effective use of technology for tracking and communication.^[35]^ These experiences collectively emphasize the necessity of preparedness, robust infrastructure, and clear communication in managing health crises effectively.^[3]^

### 2.30. Reviewing past health crises to identify best practices and lessons learned

Reviewing past health crises is critical for identifying best practices and deriving lessons that can improve future responses.^[7]^ Historical analyses of events like the SARS outbreak (2003), the Ebola epidemic (2014–2016), and the COVID-19 pandemic reveal important insights into effective crisis management and areas needing improvement.^[125]^ For example, the SARS outbreak underscored the importance of early detection and transparent communication to control the spread of infectious diseases.^[51]^ The Ebola crisis highlighted the need for robust healthcare infrastructure and international collaboration, as the lack of preparedness in affected regions led to significant delays in response.^[15]^ Similarly, the COVID-19 pandemic demonstrated the value of flexible and scalable response strategies, effective use of technology for tracking and communication, and the need for equitable healthcare access.^[29]^ These experiences collectively emphasize the importance of preparedness, communication, and collaboration in managing health crises.^[13]^

### 2.31. Applying project management retrospectives to improve future response strategies

Applying project management retrospectives to past health crises helps improve future response strategies by systematically reviewing what worked well and what did not.^[16]^ Project management retrospectives involve analyzing project outcomes, identifying successful strategies, and understanding the reasons behind any failures or shortcomings.^[6]^ This process includes gathering feedback from all stakeholders, evaluating performance against predefined metrics, and documenting lessons learned.^[16]^ For instance, after the Ebola outbreak, retrospectives led to the development of better protocols for outbreak detection and response, including the establishment of rapid response teams and improved data sharing mechanisms.^[12]^ During the COVID-19 pandemic, lessons learned from initial responses informed adjustments in policies, such as enhanced PPE guidelines and more robust testing protocols, to better manage subsequent waves of the virus.^[19]^ By systematically applying these retrospectives, emergency response strategies can be continually refined, leading to more effective management of future health crises.^[33]^

### 2.32. Funding and budgeting for emergency preparedness

Effective funding and budgeting for emergency preparedness involve detailed cost estimation and robust financial management.^[74]^ Project management techniques, such as Earned Value Management (EVM), help track expenditures and ensure alignment with budget forecasts by integrating scope, schedule, and cost.^[17]^ Securing funds through grants and public-private partnerships is essential, as seen during the COVID-19 pandemic when diversified funding sources, including emergency relief funds and private sector contributions, were crucial for scaling up healthcare infrastructure and vaccine distribution.^[18]^ Effective financial oversight, including regular audits and performance reviews, ensures that funds are used efficiently and adaptively in response to emerging needs.^[17]^

### 2.33. Project management approaches to budgeting and financial management in emergency preparedness

Project management approaches to budgeting and financial management in emergency preparedness involve detailed planning, cost estimation, and financial tracking to ensure effective resource utilization.^[64]^ Cost estimation techniques, such as analogous estimating and parametric estimating, are used to forecast financial requirements based on historical data and project parameters.^[16]^ Financial tracking is facilitated through Earned Value Management (EVM), which integrates scope, schedule, and cost to provide a comprehensive view of project performance and budget adherence.^[4]^ For instance, during the COVID-19 pandemic, project managers employed EVM to monitor expenditures related to PPE procurement, vaccine distribution, and healthcare infrastructure upgrades, enabling real-time adjustments and ensuring funds were allocated efficiently.^[12]^ Effective budgeting also requires creating contingency plans to address unforeseen expenses, ensuring that emergency responses remain flexible and adaptable to evolving situations.^[17]^

### 2.34. Strategies for securing and managing funding for public health emergencies

Securing and managing funding for public health emergencies involves leveraging multiple strategies to ensure adequate financial resources are available.^[33]^ Grant applications and public-private partnerships are crucial for obtaining initial funding.^[4]^ Grants from government agencies, such as the Department of Health and Human Services (HHS) or the World Health Organization (WHO), provide essential support for emergency preparedness initiatives.^[18]^ Public-private partnerships facilitate additional funding and resources by collaborating with businesses and nonprofit organizations.^[126]^ Effective funding management requires stringent financial oversight, including regular audits and performance reviews, to ensure funds are used appropriately and efficiently.^[3]^ For example, during the Ebola outbreak, funding was managed through a combination of international aid, government grants, and private sector contributions, demonstrating the effectiveness of a diversified funding approach.^[14]^ Similarly, the COVID-19 response saw significant investments through emergency relief funds and partnerships, which were crucial in scaling up healthcare capacities and vaccine distribution.^[19]^

### 2.35. Adapting project management software for health crisis management

Customizing Project Management Software to Meet the Specific Needs of Public Health Emergency Response.^[13]^ Customizing project management software to address the specific needs of public health emergency response involves adapting features to enhance coordination, communication, and resource management during crises.^[18]^ Standard project management tools, such as Microsoft Project or Asana, can be tailored by incorporating modules for real-time data tracking, incident management, and resource allocation.^[69]^ For example, custom dashboards and reporting features can be configured to monitor the status of emergency response activities, track resource inventory, and visualize the progress of vaccination campaigns.^[12]^ Additionally, integrating Geographic Information Systems (GIS) within project management software can improve spatial analysis and decision-making by mapping outbreak locations and resource distributions.^[51]^ This customization ensures that the software meets the unique demands of health emergencies, such as rapid information dissemination and dynamic resource allocation.^[59]^

### 2.36. Examples of software applications used in recent health crises

Several software applications have proven effective in managing recent health crises by providing specialized functionalities to support response efforts. During the COVID-19 pandemic, platforms like COVID-19 Data Tracker and Epi Info were extensively used for data collection and analysis, allowing health authorities to track infection rates, hospital capacities, and vaccination progress in real-time.^[35]^ Microsoft Teams and Zoom facilitated remote collaboration and communication among public health officials and frontline workers, ensuring continuity in strategic planning and operational coordination.^[18]^ Additionally, Tableau was used to create interactive visualizations of pandemic data, helping stakeholders understand and respond to evolving trends.^[7]^ These applications demonstrate the value of adaptable software solutions in enhancing the efficiency and effectiveness of health crisis management.^[65]^

### 2.37. Building community resilience through effective project management and public health strategies

Building community resilience involves integrating effective project management and public health strategies to prepare for and respond to emergencies.^[27]^ Project management approaches, such as risk assessment, contingency planning, and resource allocation, are critical for developing robust community preparedness plans.^[44]^ These strategies ensure that communities can anticipate potential threats, allocate resources efficiently, and implement coordinated response actions.^[16]^ For example, in response to natural disasters, project management frameworks help communities develop emergency plans, establish communication networks, and conduct training exercises, which collectively enhance their capacity to withstand and recover from crises.^[3]^ Effective public health strategies, such as promoting healthy behaviors and improving access to healthcare services, also play a crucial role in strengthening community resilience.^[13]^ For instance, initiatives aimed at increasing vaccination coverage and educating the public about disease prevention contribute to a community’s overall preparedness and ability to manage health crises.^[12]^ Project management is an important tool in health response frameworks that build preparedness, prevention, stakeholder engagement, clear goals, efficient resource use and ongoing learning, all critical to safe and cost effective response and relief operations as shown in Tables 5 and 6. It supports using risk assessment, Gantt charts and contingency planning to identify and strike tackle in advance, therefore doing away with delays in response.^[24]^ In pandemic, preparedness agile frameworks are used, which allows for reallocation of resources to running outbreaks on the go.^[16]^ It also provides a mechanism for achieving cross sectorial coordination by ensuring logistics, technologically, representatives from community and policy makers are heard.^[17]^ For instance, collaboration on a project basis during COVID-19 greatly helped speed up vaccine rollout through involving logistics companies along with healthcare providers.^[18]^ Setting vaccination targets and regularly reviewing progress to drive accountability reduces response time and is defined by project management as SMART objectives to track progress.^[19]^ It also employs tools such as Earned Value Management (EVM) which helps in the optimization of resource allocation and tracking performance parameters.^[20]^ Project management is also beneficial because of continuous learning as well as adaptation.^[20]^ In project management, iterative cycles permit mid-course corrections based on real-time data to curtail vaccine wastage, and to give higher coverage in rural areas.^[22]^ Project management principles in health response frameworks close the existing gaps by providing agility, coordination and efficiency.^[17]^ This approach does not only tackle urgent necessities but also builds the basic where we can remain resilient even in other catastrophes.^[19]^

**Table 5:** Key comparisons between traditional health response frameworks and project management

Aspect	Traditional health frameworks	Project management approach
Focus	Disease-specific, often reactive	Outcome-oriented, proactive, and adaptive^[16]^
Structure	Hierarchical and fragmented	Integrated and systematic with clear phases and milestones^[17]^
Flexibility	Limited; rigid protocols dominate	Agile, enabling adjustments based on real-time data^[19]^
Coordination	Sector-specific, with siloed activities	Cross-sector collaboration, integrating diverse stakeholders^[22]^
Monitoring and Evaluation	Post-crisis evaluation often delayed	Continuous monitoring with iterative learning loops^[20]^
Resource Allocation	Top-down, slower in emergencies	Prioritized and dynamic resource allocation^[16]^

**Table 6:** Comparison of project management approaches in crisis scenarios

Crisis type	Key challenges	Project management approach	Technologies used	Outcome/impact
COVID-19 Pandemic	Rapid virus spread, resource shortages, and vaccine distribution	Agile project management for adaptive planning and execution. Risk assessment to prioritize vaccine allocation.	AI for predictive analytics; IoT for cold chain monitoring	Accelerated vaccine deployment; improved resource allocation^[22]^
Natural Disasters	Infrastructure collapse, logistics disruptions	Waterfall model for phased recovery (immediate relief, rehabilitation, rebuilding).	Big data for disaster mapping; drones for supply delivery	Faster relief operations; effective resource utilization^[18]^
Humanitarian Crises	Displaced populations, limited coordination	Hybrid approach combining top-down strategic planning with decentralized decision-making.	Cloud-based collaboration tools; mobile health apps	Improved inter-agency coordination; enhanced healthcare access^[20]^
Chemical Spills	Environmental hazards, community displacement	Incident command system integrated with project management to streamline roles and responses.	IoT sensors for air/water quality; GIS for impact mapping	Reduced environmental damage; timely containment^[22]^
Urban Health Crises	Complex stakeholder engagement, rapid disease escalation	Lean project management to minimize waste and maximize efficiency in resource deployment.	Digital twins for scenario modeling; telemedicine for patient triage	Enhanced stakeholder collaboration; reduced patient surge in hospitals^[16]^

### 2.38. Role of community engagement in strengthening public health preparedness

Community engagement is essential for strengthening public health preparedness by fostering collaboration and trust between health authorities and residents.^[6]^ Engaging communities through public education campaigns, participatory planning, and feedback mechanisms enhances their awareness, preparedness, and willingness to follow health recommendations.^[11]^ For example, during the COVID-19 pandemic, community engagement strategies, such as outreach programs and local partnerships, were instrumental in disseminating accurate information, addressing vaccine hesitancy, and promoting public health measures.^[37]^ Effective community engagement ensures that preparedness plans are tailored to local needs and contexts, leading to more effective implementation and increased community resilience.^[3]^ By involving community members in decision-making and response efforts, public health initiatives can leverage local knowledge and resources, thereby enhancing overall preparedness and response capabilities.^[62]^

### 2.39. Ethical considerations in emergency response

#### 2.39.1. Addressing ethical issues in resource allocation, decision-making, and stakeholder engagement during health crises

**Figure 2.**
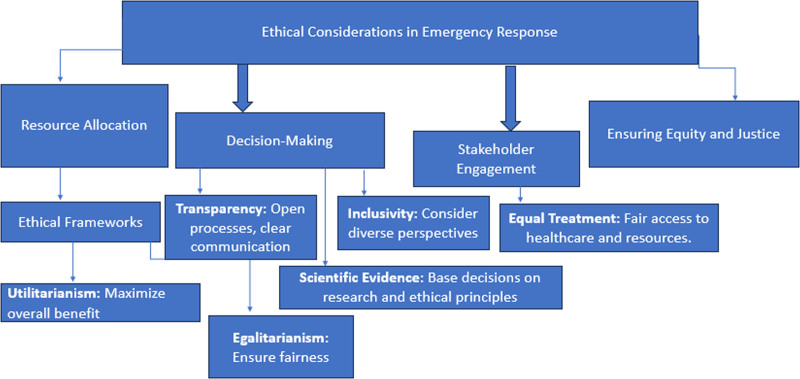
Ethical considerations in emergency response.

Addressing ethical issues in resource allocation, decision-making, and stakeholder engagement during health crises is crucial for ensuring fair and effective responses as shown in Figure 2. Resource allocation during emergencies often involves difficult decisions about prioritizing limited resources such as vaccines, treatments, and medical equipment1.^[7,10]^ Ethical frameworks, such as utilitarianism (maximizing overall benefit) and egalitarianism (ensuring fairness), guide these decisions to balance individual needs against the greater good.^[8]^ Ethical approaches such as Utilitarianism and Egalitarianism suggest distinct but sometimes similar decisions to make when there is a crisis as shown Figure 2. We then exchange the major concerns and biases utilitarianism has for how it approaches maximizing overall happiness, wellbeing, or utility for the greatest number of people or how minimizing harm or maximizing benefits is central to said approach.^[7]^ It’s practical and outcome driven, making for an extremely practical tool in crisis when decisions need to be made quickly and efficiently using limited resources.^[8]^ The strengths of utilitarianism lie in its practical and outcome oriented nature and this is why it is very much applicable in crises where quickly and resourced efficient decisions much be made.^[7]^ However, it frequently overlooks the rights of the individual or of equity in the interest of the benefit of the majority with the result that some vulnerable groups are marginalized and ethical conundrum can happen when outcomes cannot be anticipated with precision.^[8]^ Egalitarianism stresses equality, in that everyone should have easy access to resources and opportunities.^[7]^ Its focus on being fair and just enables it to work with specific marginalized or disadvantaged groups and to ensure they are not left out and to work toward long term societal cohesion by rectifying imbalances.^[7]^ However, prioritizing the equality can lead to inefficiency, delaying immediate responses in crisis, though, it may likely cause a perception of unfairness in disadvantaged groups that are disproportionately contributing to the resolution of crisis.^[9]^ In reality, the practical use of these frameworks when responding to real time crises often involves a combination of both these ways to enhance fairness and efficiency.^[10]^ For instance, towards the end of COVID-19, the approach was hybrid, prioritizing to the healthcare workers and vulnerable people (utilitarian) but also ensuring that at least the basic supply of vaccines must reach to all countries (egalitarian).^[11]^ Finally, for a more critical engagement with utilitarianism and egalitarianism in order to argue that they work together within the terrain of crisis management.^[10]^ Both these frameworks have to be weighed carefully by decision makers, requiring different context specific strategies to address immediate and long term necessities and wider societal impacts.^[11]^ There were several ethical dilemmas posed during the COVID-19 pandemic that has to be solved.^[10]^ The two main components of this process were the prioritization of limited vaccine doses to be allocated to the population through which the largest benefit to society could be achieved on a per capita basis, and the equitable allocation of the doses received.^[6]^ In the utilitarian perspective, groups that would maximize societal well being, like healthcare workers, elderly people and also people with comorbidities were prioritized, with the goal being to save as many people as possible and keep the health care system running. While this caused inequality in accessing vaccines, especially for the marginalized communities being rural populations or poor countries.^[20]^ The second challenge was to allocate resources called ventilators to hospitals. The utilitarian view prioritized patients with the best chance of survival, or otherwise with the longest remaining life expectancy, in doing so sacrificing the least number for the greatest number’s benefit.^[7]^ For instance, during the COVID-19 pandemic, ethical guidelines were developed to prioritize frontline workers and high-risk individuals for vaccination, aiming to mitigate overall societal impact while addressing immediate needs.^[6]^ During the COVID-19 peak, it was also employed, but it raised issues about discrimination and equity in relation to healthcare.^[8]^ Finally, the distribution of food and resources after natural disasters was the third challenge.^[6]^ Utilitarian approach focused on logistics and instant impact, and egalitarian approach focused on equal amount of assistance to all who suffer irrespective of their location. However, this method could have a bad impact on those in great need.^[7]^ The fourth challenge concerned the experimental drug or vaccine distribution in pandemics. Utilitarians regarded only experimental treatments as candidate for clinical trials in order to obtain reliable data that would finally benefit future populations.^[8]^ So, the egalitarian approach was that experimental treatments were given broadly, so nobody who was about to die died without a shot at survival.^[9]^ Nevertheless, it may endanger trial integrity and delay definitive results and restricted access may engender ethical considerations of the withholding of potentially lifesaving treatments.^[10]^ The fifth challenge was the evacuation of people during armed conflicts, finally the utilitarian approach favored prioritization for those with the highest contribution potential towards crisis resolution, and donating to vulnerable populations, regardless of their involvement in society was the strategy through the egalitarian approach.^[7]^ This can create conflicting priorities when trying to meet immediate survival needs and long term societal stability.^[9]^ Decision-making processes must also be transparent and inclusive, ensuring that diverse perspectives are considered, and that decisions are based on scientific evidence and ethical principles.^[20]^ Stakeholder engagement involves ethical considerations in ensuring that all affected groups, including marginalized communities, have a voice in emergency response planning and implementation.^[31]^ Effective stakeholder engagement fosters trust and cooperation, essential for successful public health interventions.^[22]^ For marginalized groups to be genuinely represented in decision making processes in emergencies, they have to be included beyond their presence and capacity built for participation.^[10]^ To help achieve this, underrepresented groups must be provided translation services, technical training, or financial assistance to participate in public health policy discussions.^[8]^ Alternatively, participatory models of decision-making, including deliberative democracy or citizen assemblies, can also directly influence outcomes.^[9]^ Tokenism should be addressed; accountability standards should be established; independent oversight should be ensured; and symbolic appointments should be prevented.^[10]^ It means representatives should be selected by and be answerable to their communities, and not either external organizations or governments in search of optics. Multi stakeholder coordination in emergencies entails conflicting priorities among stakeholders, resource inequities, and multiple cultural lenses.^[11]^ Furthermore, we need to develop funding models or partnerships that will stabilize marginalized groups capacity for engagement, including grants to local organizations.^[10]^ In coordination challenges in global emergencies there are many agencies with different mandates, so they are adopting centrally coordinated coordination hubs, coordinated but respectful of local expertise, and representation.^[8]^ These all constitute best practices for genuine representation and coordination through participatory emergency frameworks, technology for inclusivity, and equity audits.^[10]^ The representation of marginalized groups requires intentional systems for inclusion, active measures to prevent tokenism, and tools to navigate the difficulty of multi-stakeholder coordination.^[11]^ Implementing these strategies can result in more equitable, effective and inclusive emergency response.^[10]^ The successful implementation of technology in crisis management, however, depends on the active involvement of interested stakeholders from across the board.^[11]^ Of course, this interplay between technical solutions and stakeholder cooperation is necessary to guarantee both efficacy and equity in emergency response.^[11]^

### 2.40. Ensuring equity and justice in public health emergency responses

The first steps towards equity and justice in public health emergencies include endorsing the equal treatment of all persons and addressing injustices in the distribution of treatment and other resources.^[127]^ It is equally important to consider the needs of vulnerable populations within health crises as measures towards fair treatment should involve provision for low-income individuals, persons of color, and racial minorities, as well as those with preexisting medical conditions as they may face barriers to access healthcare and resources.^[125]^ Thus, public health interventions should prioritize these groups to ensure equity in tackling health issues and ensure that resources needed for handling these issues are offered based on status granted along with requirements rather than socioeconomic status.^[28]^ Distributive justice will mean that policies enforced and actions taken are in support of the rights of humans and the fight against discrimination. For example, in connection with the COVID-19 pandemic, processes associated with the equitable distribution of vaccines such as targeted advertising of vaccination, including to vulnerable populations, as well as changing the locations of vaccinations to make them more accessible.^[126]^ Incorporation of equity and justice into the disaster response plans makes it possible for public health to address systematic issues and offers a broader view of the response process.

### 2.41. Technology and innovation in urban health crisis management

#### 2.41.1. Leveraging new technologies in managing urban health crises

The application of new technologies like AI, big data, and IoT enhances the management of health emergencies in urban areas significantly.^[33]^ Machine learning is used in the area of predictive modeling and analysis for identifying locations and potential epidemics of diseases using data from past and current information. During the COVID-19 pandemic, AI models analyzed the dynamics of cases and deaths, social media activity, and mobility trends to predict surges and inform interventions.^[44]^ The application of big data in the analysis of health information involves the use of data from multiple sources, the EHRs, social media, and surveys, which are useful in providing practical insights when managing crises.^[69]^ IoT devices including health monitoring wear and environmental sensors facilitate real-time tracking of health parameters and environmental factors respectively which can in turn call for quick action in case of emerging emergencies.^[44]^ Altogether, these technologies contribute towards improving situation awareness, the distribution of resources, and decision-making in urban health emergencies.^[69]^ AI, big data, and IoT are being utilized all over for real-world intervention and monitoring in conditions of resource limits, such as healthcare delivery, monitoring, disease surveillance, and more.^[60]^ Aravind Eye Care System in India is an example with AI driven platforms using machine learning to detect diabetic retinopathy at a distant site across remote regions for early diagnosis and treatment in underserved areas.^[63]^ During the Ebola outbreak in West Africa, mobile phone records were combined with epidemiological data by big data analytics, pinpointing zones for containment and better targeting interventions and resource allocation to them.^[64]^ The mHealth initiative in sub-Saharan Africa is among the IoT enabled devices that allow patients with chronic conditions transmit their vital signs to healthcare providers using low cost wearables in mobile network to improve disease management in areas with little health care infrastructure.^[66]^ Practical barriers to implementation of these techniques include infrastructure limitations, data security and privacy concerns, costs of implementation limited skilled workforce and cultural and social barriers.^[67]^ In terms of investments on infrastructure, the solution must be made scalable using satellite internet and mobile based platforms. Building trust in digital solutions requires policy development that develops localized data protection laws. Governments, NGOs, and private companies should be partnered up to share costs and resources.^[68]^ There should be regional hubs for education and training for AI and IoT skills, and cultural sensitivity built in, through participatory design approaches, where those who will be using the solutions are involved in developing those solutions.^[68]^ In short, the promise of improving health outcomes in resource constrained settings through the use of AI, big data and the internet of things must be matched with the ability to address entrenched infrastructural, economic and social barriers.^[69]^ With these technologies, these practical solutions with the assistance of local partnerships can be deployed to effectively bridge the gap in healthcare equity.^[70]^

### 2.42. Innovations in project management tools to enhance emergency response capabilities

Technology is a significant determinant of the enhancement of first response systems and relates to the improvement of the project management systems that are applied in emergencies.^[9]^ Today’s integrated project management systems offer such options as real-time work contact during 24 hours, data visualization, and certain preventive-automated options for emergency cases. Software applications include Monday. com and Smartsheet have customizable dynamic dashboards and tracking systems for complex response actions and resources.^[11]^ Moreover, technological innovations in geospatial analysis and drones produce a significant change to disaster management in terms of rapid mapping of affected areas and timely delivery of products.^[2]^ In natural disasters, there were cases of using drones with cameras and sensors to assess the level of damage, deliver medicals, and aid in search and rescue.^[5]^ It is noteworthy, along with current developments in project management and state-of-the-art technology, that the flexibility and effectiveness of operations relating to emergency responses have increased significantly.

## 3. Conclusion

The process of managing of emergency response systems in urban health emergencies requires a systems approach that integrates concepts from project management, technologies, ethical considerations, stakeholder participation, and continuous improvement. The alignment of these particular areas enables the enhancement of readiness as well as emergency response capacities to public health authorities when managing health emergencies for more effective solutions to affected groups or communities.

## 4. Recommendations

Upon evaluating the public health readiness and response measures, several important recommendations can be highlighted: It is crucial to incorporate project management frameworks with public health initiatives, to enhance the outcome of public health interventions. This integration involves the embedding of health emergency-specific features in the project management software that may include; the ability to monitor data, the ability to assess the risks associated with a particular case, and the ability to award resources. Recent advancements in technologies that include artificial intelligence, big data, and the internet of things help to boost the accuracy of the predictors, real-time monitoring, and decision-making processes. Such integrated technologies should be a priority for public health organizations to ensure a much more policy-coordinated and efficient response during emergent conditions. Ethical considerations of the principal type provide a noble and reasonable basis through which to promote fair efficient and timely response to emergencies. Some of the more important objectives include the need to define and make ethical guidelines for budgeting, decision-making on resources to be allocated, and the involvement of stakeholders. This includes the provision of equal opportunities for the supply of resources for the oppressed groups and the promotion of political oblivion in the assessment of decisions. It is for this reason that drawing up and enforcing guidelines for ethical decision-making can help to successfully navigate the complex realities of the existence of health threats, and ensure that actions taken are equal for all the contestants of the games. There is also the concept of ongoing training and awareness raising among the decision makers also helps them to stay ethical even in emergencies. Two bodies of literature are relevant to the concept of effective participation to increase the community’s level of preparedness for disease outbreaks: Laypersons should consequently be intentionally involved in planning and responding by authorities, and their local intelligence and self-confidence enhanced. Information given through the development of public education campaigns, planning with participation, and ensuing contact could achieve this. Improving community engagement improves the capability of programs to respond to disasters not only in functionality but also in the protection and recovery systems of the affected societies. It seems advisable to ensure that preparation plans contain and reflect all needed employment for making improvements to total preparedness. Advanced technologies and project management tools enhance skills in emergency response when in actual use in a project. PM and other modes of technology should be used to source for complex equipment such as integrated project management infrastructure, unmanned aerial vehicles, and geospatial analysis technology. In addition, such advancements can improve the visualization of data in near real-time, resource tracking, and awareness of the operational environment during emergency conditions. With such technologies, the health authorities would be in a position to improve the manner they respond to emergencies and adapt to new situations in the HELios system as well as in other health system reform needs. Training and upgrading of readiness processes is crucial especially because prior learning helps create subsequent improvement of health emergences. To assess the effectiveness of the responses and to understand the need for changes, improvements, refinements, and adjustments, there must be solid monitoring and evaluation systems developed and put in place by the public health organizations. Continuing to hold retrospectives and using what has been learned from previous incidents continues to improve the strategies that are employed, as well as increasing the capacity of the bigger organizational response. Appropriate and sustainable practices of learning and adaptation have to be consistently promoted to sustain and enhance the responsiveness of public health systems to challenges in the future. The above suggestions aim at the overall improvement of the general usability of public health emergency management through the incorporation of improved technologies, addressing of ethical issues, increased community engagement, and always continued improvement.

## Acknowledgments

We are grateful to Kampala International University Uganda for its support.

## Author contributions

**Conceptualization:** Tom Nyamboga Ongesa, Okechukwu Paul-Chima Ugwu, Esther Ugo Alum.

**Methodology:** Chinyere N. Ugwu, Esther Ugo Alum, Mariam Basajja, Jovita Nnenna Ugwu, Michael Ben Okon, Regina Idu Ejemot-Nwadiaro.

**Supervision:** Tom Nyamboga Ongesa, Okechukwu Paul-Chima Ugwu, Esther Ugo Alum, Val Hyginus Udoka Eze, Mariam Basajja, Fabian C. Ogenyi, Regina Idu Ejemot-Nwadiaro.

**Validation:** Tom Nyamboga Ongesa, Okechukwu Paul-Chima Ugwu, Chinyere N. Ugwu, Val Hyginus Udoka Eze, Mariam Basajja, Jovita Nnenna Ugwu, Fabian C. Ogenyi, Michael Ben Okon.

**Visualization:** Tom Nyamboga Ongesa, Okechukwu Paul-Chima Ugwu, Chinyere N. Ugwu, Esther Ugo Alum, Val Hyginus Udoka Eze, Mariam Basajja, Jovita Nnenna Ugwu, Fabian C. Ogenyi, Michael Ben Okon, Regina Idu Ejemot-Nwadiaro.

**Writing – original draft:** Tom Nyamboga Ongesa, Okechukwu Paul-Chima Ugwu, Chinyere N. Ugwu, Esther Ugo Alum, Val Hyginus Udoka Eze, Mariam Basajja, Jovita Nnenna Ugwu, Fabian C. Ogenyi, Michael Ben Okon, Regina Idu Ejemot-Nwadiaro.

**Writing – review & editing:** Tom Nyamboga Ongesa, Okechukwu Paul-Chima Ugwu, Chinyere N. Ugwu, Esther Ugo Alum, Val Hyginus Udoka Eze, Mariam Basajja, Jovita Nnenna Ugwu, Fabian C. Ogenyi, Michael Ben Okon, Regina Idu Ejemot-Nwadiaro.
